# Purinergic receptors are involved in tooth-pulp evoked nocifensive behavior and brainstem neuronal activity

**DOI:** 10.1186/1744-8069-6-59

**Published:** 2010-09-22

**Authors:** Kazunori Adachi, Kohei Shimizu, James W Hu, Ikuko Suzuki, Hiroshi Sakagami, Noriaki Koshikawa, Barry J Sessle, Masamichi Shinoda, Makiko Miyamoto, Kuniya Honda, Koichi Iwata

**Affiliations:** 1 Department of Diagnostic & Therapeutic Sciences, Meikai University School of Dentistry, 1-1 Keyaki-dai, Sakado, 350-0283, Japan; 2 Department of Pharmacology, Nihon University School of Dentistry, 1-8-13 Kanda-Surugadai Chiyoda-ku, Tokyo, 101-8310, Japan; 3 Division of Oral and Craniomaxillofacial Research, Dental Research Center, Nihon University School of Dentistry, 1-8-13 Kanda-Surugadai Chiyoda-ku, Tokyo, 101-8310, Japan; 4 Department of Endodontics, Nihon University School of Dentistry, Nihon University School of Dentistry, 1-8-13 Kanda-Surugadai Chiyoda-ku, Tokyo, 101-8310, Japan; 5 Department of Oral Physiology, Faculty of Dentistry, University of Toronto, 124 Edward Street, Toronto, M5G 1G6, Canada; 6 Department of Physiology, Nihon University School of Dentistry, Nihon University School of Dentistry, 1-8-13 Kanda-Surugadai Chiyoda-ku, Tokyo, 101-8310, Japan; 7 Division of Functional Morphology, Dental Research Center, Nihon University School of Dentistry, Nihon University School of Dentistry, 1-8-13 Kanda-Surugadai Chiyoda-ku, Tokyo, 101-8310, Japan; 8 Department of Anesthesiology, Nihon University School of Dentistry, Nihon University School of Dentistry, 1-8-13 Kanda-Surugadai Chiyoda-ku, Tokyo, 101-8310, Japan; 9 Oral and Maxillofacial Surgery Nihon University School of Dentistry, Nihon University School of Dentistry, 1-8-13 Kanda-Surugadai Chiyoda-ku, Tokyo, 101-8310, Japan

## Abstract

**Background:**

To evaluate whether P2X receptors are involved in responses to noxious pulp stimulation, the P2X_3 _and P2X_2/3 _receptor agonist α,β-methyleneATP (α,β-meATP) was applied to the molar tooth pulp and nocifensive behavior and extracellular-signal regulated kinase (ERK) phosphorylation in trigeminal spinal subnucleus caudalis (Vc), trigeminal spinal subnucleus interpolaris (Vi), upper cervical spinal cord (C1/C2) and paratrigeminal nucleus (Pa5) neurons were analyzed in rats.

**Results:**

Genioglossus (GG) muscle activity was evoked by pulpal application of 100 mM α,β-meATP and was significantly larger than GG activity following vehicle (phosphate-buffered saline PBS) application (p < 0.01). The enhanced GG muscle activity following 100 mM α,β-meATP was significantly reduced (p < 0.05) by co-application of 1 mM TNP-ATP (P2X_1_, P2X_3 _and, P2X_2/3 _antagonist). A large number of pERK-LI cells were expressed in the Vc, Vi/Vc, C1/C2 and Pa5 at 5 min following pulpal application of 100 mM α,β-meATP compared to PBS application to the pulp (p < 0.05). The pERK-LI cell expression and GG muscle activity induced by 100 mM α,β-meATP pulpal application were significantly reduced after intrathecal injection of the MAPK/ERK kinase (MEK) inhibitor PD 98059 and by pulpal co-application of 1 mM TNP-ATP (p < 0.05).

**Conclusions:**

The present findings suggest that activation of P2X_3 _and P2X_2/3 _receptors in the tooth pulp is sufficient to elicit nociceptive behavioral responses and trigeminal brainstem neuronal activity.

## Background

Adenosine 5'-triphosphate (ATP) is considered a neuro-modulator in primary afferent neurons. Release of ATP from sympathetic nerve terminals, endothelial, Merkel or tumor cells is known to be involved in excitation of unmyelinated primary afferent neurons [1]. One of ATP receptors activated by ATP binding is the P2X family of ATP receptors; it has been classified into seven subtypes, P2X_1-7 _(for review, see [2-8]). All of them, except the P2X_7 _receptor, are expressed in various primary sensory neurons including tooth pulp neurons [9-13]. In particular, the P2X_3 _homomeric and P2X_2/3 _heteromeric receptors have been associated with peripheral nociceptive mechanisms, since these subtypes occur in a subset of putative nociceptive sensory neurons [10-12,14-16], and their activation produces nocifensive behavior that can be attenuated by peripheral [17-20] or central [16,21] administration of P2X_3,2/3 _receptor antagonists. Also activation of pulpal P2X_3,2/3 _receptors produces central sensitization in functionally identified nociceptive brainstem neurons in the trigeminal subnucleus caudalis (Vc) [16,22]. Pulpal administration of capsaicin, mustard oil or other inflammatory substances are known to strongly activate Vc neurons suggesting that TRPV1 and TRPA1 receptors or other receptors related to inflammation are involved in tooth pulp pain [23,24]. It is very important to know which receptors in the pulpal nerve terminals are involved and how these receptors mediate tooth pulpal pain, in order to understand the neuronal mechanisms of tooth pulp inflammatory pain. ATP is known as one of the important neuro-modulators involved in tooth pulp inflammatory pain [9-12]. However, how ATP is involved in pulpal pain during inflammation remains unclear. We thus introduced α,β-meATP as a P2X_2/3 _receptor agonist to exclude the effect of substances other than ATP.

The phosphorylated extracellular signal-regulated kinase (pERK), one of the mitogen-activated protein kinases (MAPKs), has been recognized as a marker of activation of spinal dorsal horn (DH) neurons following a variety of noxious stimuli applied to peripheral tissues [25,26]. ERK can be phosphorylated in Vc and upper cervical spinal cord (C1/C2) neurons within 10 min following peripheral noxious stimulation, and the number of pERK-positive neurons progressively increases as stimulus intensity is increased. We have recently reported that pERK-positive neurons are expressed in Vc and the Vc/C1 transition region as well as in the transition zone between the trigeminal spinal subnucleus interpolaris (Vi) and Vc (Vi/Vc) following noxious stimulation of orofacial regions including the tooth pulp [23,27,28]. These findings suggest that the phosphorylation of ERK is strongly correlated with excitation of Vi/Vc, Vc and C1/C2 neurons following noxious stimulation of the orofacial region.

Given these various findings, it is of interest to study the distribution pattern of Vi/Vc, Vc and C1/C2 nociceptive neurons that can be activated by specific stimulation of tooth pulp purinergic receptors, in order to clarify the involvement of these receptors in tooth pulp-induced V central sensitization. However, the distribution pattern of the Vc, Vi/Vc and C1/C2 neurons sensitized by the activation of tooth pulp purinergic receptors is unknown. Therefore, we have investigated the effects of pulpal application of purinergic receptor agonist on the expression of pERK in Vi/Vc, Vc and C1/C2 neurons and if their activation is associated with nocifensive behavior reflected as an increase in electromyographic (EMG) activity of masticatory muscles. Some of the data have been published in abstract form [29].

## Methods

### Animals

Seventy-five male Sprague-Dawley adult rats (300-360 g) were used in this study. The rats were housed in individual cages (27 × 45 × 20 cm) in a temperature-controlled (21 ± 1°C) and humidity-controlled (50 ± 5%) room under a 12 hr light/dark cycle (lights on at 07:00 am) with free access to food and water. All procedures were approved by the University of Toronto Animal Care Committee in accordance with the regulations of the Ontario Animal Research Act (Canada) and animal experimentation committee in Nihon University School of Dentistry.

### Drugs

The drugs used were α,β-methylene adenosine 5'-triphosphate, (α,β-meATP, Sigma-Aldrich, St. Louis, MO, USA), a P2X_1_, P2X_3 _and, P2X_2/3 _agonist, and 2',3'-O-(2,4,6-trinitrophenyl) adenosine 5'-triphosphate (TNP-ATP, Sigma-Ardrich), a P2X_1_, P2X_3 _and, P2X_2/3 _antagonist; both were dissolved in 1 mM phosphate-buffered saline (PBS; pH = 7.4). The agonist and antagonist (or vehicle PBS) were cocktailed for combined application. The solution (approximately 0.2 μl) was applied to the tooth pulp by a soaked dental paper point. The mitogen-activated extracellular signal-regulated kinase (MEK) inhibitor PD 98059 (Calbiochem, La Jolla, CA, USA) was dissolved into 10% Dimethyl sulfoxide (DMSO) and the solution placed into a mini-osmotic pump (model 2001, Durect Co. Cupertino, CA, USA) that was implanted under the skin of rat neck, so that PD 98059 could be continuously (1 μl/h) diffused intrathecally for one week before EMG and/or immunohistochemical experiment [27,30,31].

### Electromyographic (EMG) recording

**Figure 1:**
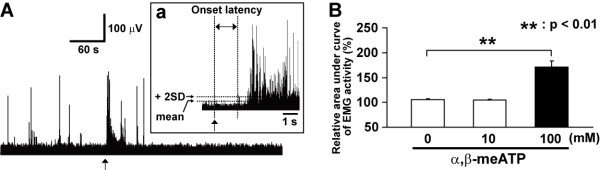
**GG muscle EMG activity induced by pulpal application of αβ-meATP**. A: GG muscle EMG activity induced by pulpal application of α,β-meATP (100 mM). The arrow indicates the timing for drug application. Aa: Onset latency was determined from the time lag between the drug application and onset of EMG activity above 2 SDs. B: Relative area under curve of genioglossus muscle EMG activity induced by pulpal application of α,β-meATP. The data were obtained during 1 min after pulpal application of α,β-meATP (0, 10 and 100 mM). Data were analyzed using one-way ANOVA with repeated measures followed by Bonferroni multiple comparison test (**: *P *< 0.01).

Anesthesia was maintained by 1-2% halothane (Halocarbon Products Corp., River Edge, NJ, USA) for the implantation of bipolar EMG electrode wires and for preparation of an occlusal cavity in the right maxillary first molar [23]. During the period of the subsequent experiment, the concentration of halothane was reduced to < 0.9%. Body temperature was maintained at 37-38°C by a feedback-controlled blanket (Model 73A, YSI, Yellow Springs, OH, USA). Heart rate was continuously monitored and maintained at physiological levels of 330-430 beats/min. Pairs of EMG electrodes (40-gauge, Teflon^®^-insulated stainless wires; Cooner wire, Chatsworth, CA, USA) were implanted into the right masseter (MA), anterior digastric (AD) and genioglossus (GG) muscles to record any tooth pulp-evoked muscle activities (Fig. 1 and Table 1), as previously described [24]. The placement of EMG electrodes was confirmed by muscle twitches induced by electrical stimulation of the EMG electrodes (12 × 0.2 ms pulses, 333 Hz, 100-200 μA).

**Table 1:** Incidence, onset latency and duration of muscle activities induced by chemical administration to the tooth pulp

	PBS		α,β-meATP (10 mM)		α,β-meATP (100 mM)		α,β-meATP (100 mM) + TNP-ATP (0.1 mM)		α,β-meATP (100 mM) + TNP-ATP (1 mM)	
	
Muscle	Incidence	Latency (s)	Incidence	Latency (s)	Incidence	Latency (s)	Incidence	Latency (s)	Incidence	Latency (s)
GG	2/7	1.71 ± 0.46	2/4	2.13 ± 0.33	10/10	2.15 ± 0.53	4/4	3.56 ± 1.07	5/5	1.24 ± 0.46
AD	2/7	1.70 ± 0.45	2/4	1.80 ± 0.00	10/10	1.85 ± 0.46	2/4	3.76 ± 0.96	2/5	6.21 ± 2.42
MA	0/7	-	0/4	-	2/10	1.33 ± 0.00	0/4	-	0/5	-

		**Duration (s)**		**Duration (s)**		**Duration (s)**		**Duration (s)**		**Duration (s)**

GG	2/7	0.05 ± 0.00	2/4	1.87 ± 1.85	10/10	31.78 ± 8.04	4/4	2.30 ± 1.25 *	5/5	4.86 ± 2.10 *
AD	2/7	0.03 ± 0.02	2/4	0.03 ± 0.01	10/10	1.12 ± 0.52	2/4	0.02 ± 0.00	2/5	0.03 ± 0.01
MA	0/7	-	0/4	-	2/10	1.53 ± 0.19	0/4	-	0/5	-

GG, ipsilateral genioglossus muscle; AD, ipsilateral anterior digastric muscle; MA, ispilateral masseter muscle. Latency and duration were expressed as mean ± SEM. *: P < 0.05 (vs 100 mM α,β-meATP, Bonnferroni multiple t-test).

The occlusal cavity (< 0.7 mm in diameter and 0.6-0.8 mm in depth) in the molar tooth pulp was prepared by a dental drill (at low speed) carrying a carbide bur (0.5 mm in diameter). During drilling, the molar occlusal surface was cooled by a cotton pellet soaked with cool saline. Then, the cavity was immediately filled with a small piece of cotton pellet soaked with isotonic saline. Any exposed pulp manifesting intense bleeding was considered to be severely injured and was excluded from the subsequent experiments. To allow for unrestricted orofacial muscle movements, the rat's head was held upright, with the rat in the prone position, by means of metal rods which were fixed on the skull with stainless-steel screws and dental acrylic. The mandible could thus be gently pulled down to obtain enough space to access the maxillary molar, so that prior to drug application the saline soaked cotton pellet could be removed from the cavity and a segment of dental paper point soaked with drugs or vehicle could be applied into the tooth pulp through the cavity and filled with dental temporary cement (Cavit^®^, 3 M, St. Paul, MN, USA) [16,24]. Either 10 mM (n = 2) or 100 mM (n = 8) α,β-meATP, 1 mM TNP-ATP (n = 2), 100 mM α,β-meATP with 0.1 mM TNP-ATP (n = 3), 100 mM α,β-meATP with 1 mM TNP-ATP (n = 3) or PBS (n = 5) was applied to the cavity. Since two of rats in each group were received drug application to both side of maxillary first molar, respectively, the sample number of each group for data analysis was 10 (n = 4) or 100 (n = 10) mM α,β-meATP, 1 mM TNP-ATP (n = 4), 100 mM α,β-meATP with 0.1 mM TNP-ATP (n = 5), 100 mM α,β-meATP with 1 mM TNP-ATP (n = 5) and PBS (n = 7) (Table 1). An additional 6 rats pretreated with PD 98059 also received 100 mM α,β-meATP. Muscle activities were continuously recorded before, during and after PBS or α,β-meATP administration to the pulp, and the mean value of EMG activity was calculated at every 1 min before and after drug application.

In order to test whether the pulpal application of the P2X_3 _and P2X_2/3 _receptor agonist α,β-meATP causes an increase in the EMG activity in MA, AD or GG under our anesthetic condition, noxious mechanical stimulus was applied to the facial skin and the threshold intensity for evoking EMG activity in each muscle was measured in another 6 rats. Animals were placed in a stereotaxic apparatus immediately after EMG wire implantation and filaments of varying forces were applied to the surface of the left temporomandibular joint (TMJ) region in ascending order. Since it has reported that the TMJ is an appropriate region for stimulation to determine the reflex threshold (reflex sensitivity) of each orofacial muscle [32,33]. Each filament was applied for a maximum of 10 s and for 5 stimulus trials. When the EMG activity induced in each muscle by such stimulation exceeded mean baseline amplitude over +2SD after drug application was considered to be positive response and the force which induced 3 positive responses of 5 trials was defined as the threshold for that muscle.

As previously described [34,35], the EMG activity was amplified (gain 1000×, filtered [bandpass 100-5 kHz]; model 1700, A-M systems, Carlsborg, WA, USA) and digitized (10 × 10^3 ^samples/s) by an A/D converter and Spike2 software (CED 1401 plus, Cambridge Electronic Design, Cambridge, UK) with a PC and continuously recorded for more than 40 min during mechanical stimulation or application of drugs, then was processed off-line analysis. For each contralateral muscle of pulpal drug(s) application, EMG activities were rectified and digitally smoothed (moving average, 4-ms window). The mean baseline activity was obtained from the 10 ms period before drug application. When the EMG activity exceeded mean baseline amplitude over +2SD after drug application, it was defined as a positive response and its onset latency and duration were determined. The area under the curve (AUC) of EMG activity was measured for 10 min before and after drug application (Fig. 1 and Table 1).

### pERK immunohistochemistry

For immunohistochemical experiments, 42 rats were divided to 7 groups (n = 6/group). Under general anesthesia by 1-2% halothane, the pulp of the right maxillary first molar was exposed, and drugs (PBS, 100 mM α,β-meATP, 1 mM TNP-ATP and 1 mM TNP-ATP + 100 mM α,β-meATP) were applied to the cavity as described above. To test whether jaw opening procedure caused an activation of TMJ noxious afferents affecting Vc neuronal excitability lidocaine (50 μl) was injected into the ipsilateral TMJ capsule in the halothane-anesthetized rats. Thirty min after TMJ injections, rats received 100 mM α,β-meATP pulpal application. After appropriate survival times (5 min), rats were perfused transcardially with 500 ml 0.9% saline followed by 500 ml 4% paraformaldehyde in 0.1 M phosphate buffer (PB, pH 7.4). The same procedure was carried out on the animals which received infusion pump implantation one week before and those were received 100 mM α,β-meATP pulpal application. The whole brain including medulla and upper cervical cord was removed and post-fixed in the same fixative for three days at 4°C. The tissues were then transferred to 20% sucrose (w/v) in PBS for several days for cryoprotection. Thirty-micron-thick sections were cut from the brain stem including trigeminal spinal subnucleus caudalis and upper cervical spinal cord with a freezing microtome, and every 4th section was collected in PBS. Free-floating tissue sections were rinsed in PBS, 10% normal goat serum in PBS for 1 h, and then incubated in rabbit anti-phospho-p44/42 MAP kinase antibody (1:1000, Cell Signaling Technology Inc., Danvers, MA, USA) for 72 h at 4°C. Next, the sections were incubated in biotinylated goat anti-rabbit IgG (1:600; Vector Labs, Burlingame, CA, USA) for 2 h at room temperature. After washing, the sections were incubated in peroxidase-conjugated avidin-biotin complex (1:100; Vector Labs) for 2 h at room temperature. After washing in 0.05 M Tris buffer (TB), the sections were incubated in 0.035% 3,3'-diaminobenzidine-tetra HCl (DAB, Sigma-Ardrich), 0.2% nickel ammonium sulfate and 0.05% peroxide in 0.05 M TB (pH 7.4). The sections were washed in PBS, serially mounted on gelatin-coated slides, dehydrated in alcohols and cover slipped. The pERK-LI cells were drawn 1 or more investigators under blind design to avoid inter experimenter error under a light microscope using camera-lucida drawing tube. The number of pERK-LI cells was counted from every eighth section. The total number of pERK-LI cells from three of every section was calculated, and the mean number of pERK-LI cells (three sections/rat) was obtained from each animal in order to avoid the variability of the number of immunoreactive neurons in each section. Double-immunofluorescence histochemistry was also used to determine whether the pERK-LI cells expressed a neuronal label NeuN in the Vc and C1-C2 of 100 mM α,β-meATP-treated rats.

### Statistical analysis

Statistical analysis was performed using one-way analysis of variance followed by Bonferroni multiple comparison tests. Student's and Paired *t*-test were used for comparison between two groups when appropriate. Differences were considered significant at *P *< 0.05. Results are presented as mean ± SEM.

## Results

### EMG activities following pulpal P2X receptor stimulation

A number of studies have reported that the transient increase in the MA and AD muscle activities can be recorded following noxious stimulation of the orofacial region in anesthetized rats [23,24,36,37]. To define threshold intensity to evoke EMG activity in MA, AD or GG muscles under the present anesthetic condition, noxious mechanical stimulus was applied to the facial skin to measure mechanical threshold evoking EMG activity in MA, AD and GG muscles. The mechanical threshold evoking EMG activity was significantly lower in GG compared with AD and MA (GG: 54.3 ± 5.7 g, AD: 126.7 ± 39.6 g, MA: 166.7 ± 33.7 g, respectively, *F*(2,15) = 4.15, *P *< 0.05; ANOVA followed by Bonferroni multiple *t*-test). Incidence, onset latency and duration of GG, AD and MA activity following pulpal application of α,β-meATP and/or TNP-ATP are shown in Table 1. Consistent EMG activity could only be recorded in GG following pulpal application of 100 mM α,β-meATP (Table 1). Thus, GG activity was analyzed as the indicator of the reflex response following stimulation of the tooth pulp in the present study.

Typical GG muscle activity and mean EMG activity are illustrated in Fig. 1A. A rapid and significant increase in EMG activity could be recorded in GG (2.15 + 0.53 s) after 100 mM α,β-meATP application, but not 10 mM α,β-meATP, application compared to PBS administration (*F*(2,20) = 14.09, *P *< 0.01, ANOVA followed by Bonferroni multiple *t*-test) (Table 1, Fig. 1A, B).

### pERK-LI neurons following P2X receptor stimulation

**Figure 2:**
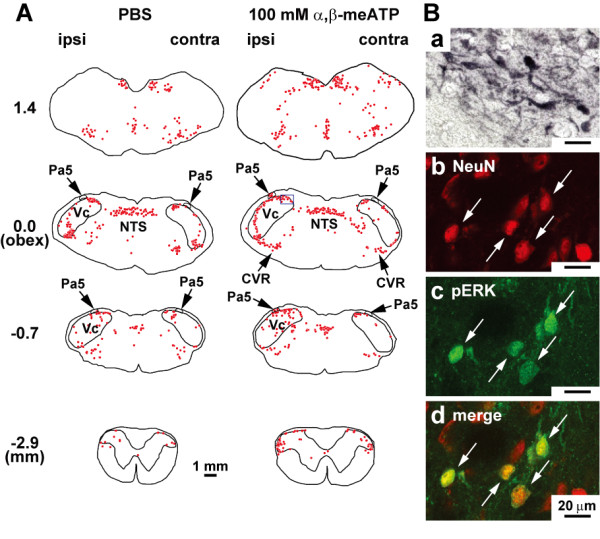
**pERK-LI cells in the bilateral medulla and upper cervical cord following pulpal administration of PBS or α, β-meATP**. A: Camera-lucida drawings of pERK-LI cells in the ipsilateral (ipsi) and contralateral (cont) medulla and upper cervical cord following pulpal administration of PBS or α,β-meATP (100 mM). Vc; trigeminal spinal subnucleus caudalis. NTS; nucleus tractus solitarii. CVR; caudal ventral reticular nucleus. Ba: Photomicrograph of pERK-LI cells in Vc. Bb: NeuN-Li cells (red) in Vc, Bc: pERK-Li cells (green) in Vc, Bd: Double labeling of pERK (green) and NeuN (red) in Vc. Arrows indicate both NeuN and pERK double-labeled cells in Vc.

**Figure 3:**
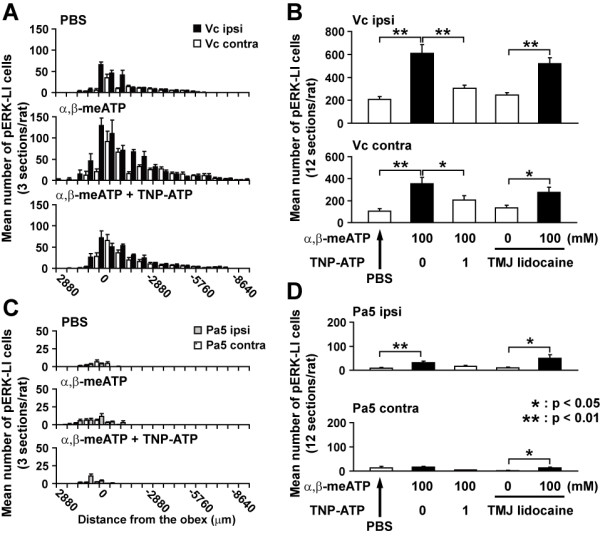
**Effect of TNP-ATP on mean number of pERK-LI cells in the bilateral medulla and upper cervical cord following pulpal αβ-meATP administration**. Mean number and rostro-caudal distribution of pERK-LI cells in ipsilateral (ipsi) and contralateral (cont) Vc and C1/C2 (A), and Pa5 (C) region induced by pulpal drug application (PBS, α,β-meATP and α,β-meATP +TNP-ATP). Mean number of pERK-LI cells in ipsilateral and contralateral Vc and C1/C2 (B), and Pa5 (D) region induced by pulpal drug application (PBS, α,β-meATP and α,β-meATP +TNP-ATP) or α,β-meATP application following lidocaine injection into TMJ. Data were analyzed using one-way ANOVA with repeated measures followed by Bonferroni multiple comparison test (*: *P *< 0.05, **: *P *< 0.01).

A number of pERK-like immunoreactive (LI) cells were expressed in Vc, Vi/Vc, C1/C2, paratrigeminal nucleus (Pa5), caudal ventral reticular nucleus (CVR) and nucleus tractus solitarii (NTS) 5 min after PBS or 100 mM α,β-meATP application into the right maxillary first molar pulp (Fig. 2A). The pERK-LI cells showed round soma and many fibers distributed around the soma as illustrated in Fig. 2Ba; all of them showed NeuN immunoreactivity (e.g. as shown by the arrows in Fig. 2Bb, Bc and 2Bd), suggesting that all pERK-LI cells observed in the present study could be classified as neurons. The rostro-caudal distribution of pERK-LI cells and the mean number of pERK-LI cells are illustrated in Fig. 3. In all of these areas, pERK-LI cells were observed in both ipsilateral and contralateral sides to the α,β-meATP application to the pulp. The number of pERK-LI cells was larger in Vc and C1/C2 regions compare to those in the contralateral side to injection. The pERK-LI cells were distributed in Vc and C1/C2 rostro-caudally, with a peak at about 0.0-0.7 mm caudal to the obex (Fig. 3A). At the obex level, many pERK-LI cells were observed from the dorsal to ventral portion of the Vc. On the other hand, there were no obvious peaks in the rostro-caudal distribution of pERK-LI cells in Pa5 (Fig. 3B). While pERK-LI cells were also expressed after PBS injection into the tooth pulp, pulpal application of 100 mM α,β-meATP produced a significantly larger number of pERK-LI cells in the Vc and Pa5 compared to PBS-injected rats (Vc ipsi: *F*(3, 23) = 20.08, *P *< 0.01; Vc cont: *F*(3, 23) = 11.70, *P *< 0.01; Pa5 ipsi: *F*(3, 23) = 7.49, *P *< 0.01; Pa5 cont: *F*(3, 23) = 3.93, *P *< 0.05, ANOVA; Vc ipsi, Vc cont and Pa5 ipsi: *P *< 0.01, respectively, Bonferroni multiple comparison test, Fig. 3C and 3D).

In order to elucidate whether the sensory input from the TMJ could have influenced the Vc and C1/C2 neuronal activity since the mouth had to be widely opened to allow access to the pulp, we also analyzed pERK-LI cell expression in Vc and Pa5 following lidocaine injection into the TMJ region bilaterally. No differences were observed in the number of pERK-LI cells in Vc and Pa5 between TMJ-anesthetized rats and intact rats following 100 mM α,β-meATP into the tooth pulp (data not shown).

### Effect of TNP-ATP and PD98059 administration on pERK-LI cell expression

The number of pERK-LI cells was significantly larger in both sides of Vc and in ipsilateral Pa5 following pulpal application of α,β-meATP compared to PBS-injected rats (Fig. 3C). The numbers of pERK-LI cells in Vc and Pa5 were significantly smaller in those rats receiving co-application of 1 mM TNP-ATP (P2X_1_, P2X_3 _and, P2X_2/3 _antagonist) with 100 mM α,β-meATP compared with those receiving 100 mM α,β-meATP application alone (see above for detail of ANOVA; Vc ipsi, Vc cont and Pa5 ipsi: *P *< 0.01, respectively; Pa5 cont: *P *< 0.05, Bonferroni multiple *t*-test, Fig. 3C and 3D).

**Figure 4:**
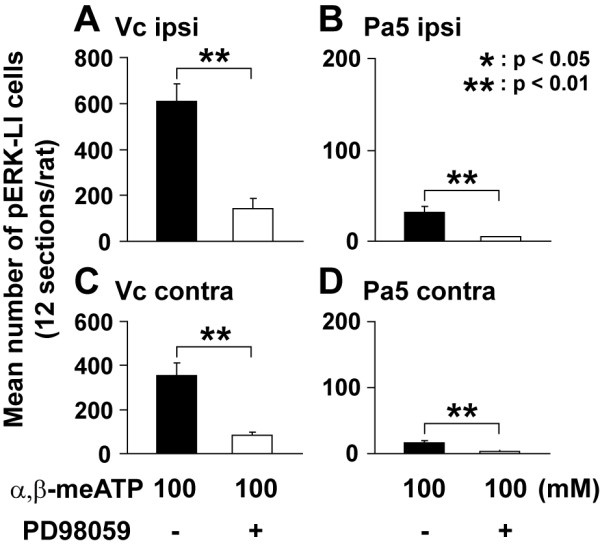
**Mean total number of pERK-LI cells in ipsilateral (ipsi) and contralateral (cont) Vc and C1/C2 (A, C), and Pa5 (B, D) region induced by pulpal application of α,β-meATP (100 mM) following intrathecal administration of PD98059**. Data were analyzed using one-way ANOVA with repeated measures followed by Bonferroni multiple comparison test (*: *P *< 0.05, **: *P *< 0.01).

Furthermore, the numbers of pERK-LI cells on both sides of Vc and Pa5 were significantly smaller in those rats receiving pretreatment of intrathecal administration of PD 98059 and α,β-meATP pulpal application compared to those receiving 100 mM α,β-meATP pulpal application alone (Vc ipsi: *P *< 0.01, Vc cont: *P *< 0.01, Pa5 ipsi: *P *< 0.01, Pa5 cont: *P *< 0.05, Student's *t*-test, Fig. 4).

### Effect of TNP-ATP and PD98059 on GG EMG activity

**Figure 5:**
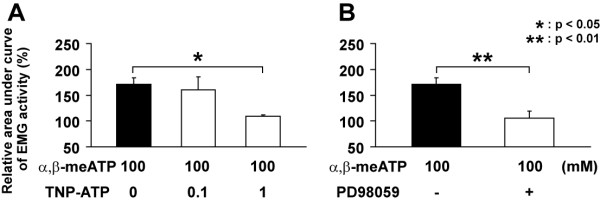
**Relative area under curve of GG muscle EMG activity induced by pulpal application of α,β-meATP (100 mM) together with TNP-ATP (A), or following intrathecal administration of PD98059 (B)**. The data were obtained during 1 min after pulpal application of α,β-meATP. Data were analyzed using one-way ANOVA with repeated measures followed by Bonferroni multiple comparison test (*: *P *< 0.05).

Onset latency of GG muscle activity was not significantly different between α,β-meATP-injected and α,β-meATP +TNP-ATP-injected rats (Table 1). On the other hand, the duration of EMG activity was significantly longer in GG following pulpal administration of 100 mM α,β-meATP (*F*(2,16) = 7.62, *P *< 0.01; ANOVA followed by Bonferroni multiple *t*-test) compared to that following co-administration of 100 mM α,β-meATP with 0.1 mM (*P *< 0.05) or 1 mM (*P *< 0.01) of TNP-ATP. The GG activity following 10 mM α,β-meATP application was significantly smaller in those rats receiving pulpal co-application of 1 mM TNP-ATP than that in rats receiving α,β-meATP application alone (*F*(2,18) = 4.69, *P *< 0.05, ANOVA followed by Bonferroni multiple *t*-test). However, we could not observe any significant effect or GG activity of pulpal co-application of 0.1 mM TNP-ATP with 100 mM α,β-meATP compared to 100 mM α,β-meATP application alone (Fig. 5A). GG EMG activity following 100 mM α,β-meATP application was significantly smaller in those rats receiving intrathecal administration of PD98059 compared with that of the rats receiving α,β-meATP pulpal application alone (*P *< 0.01, Student's *t*-test, Fig. 5B).

## Discussion

Both P2X_3 _homomultimer and P2X_2/3 _heteromultimer receptors have been reported in a variety of tissues, including tooth pulp [9-13], stimulation of which causes a barrage of action potentials in tooth pulp afferents that are conveyed to the rat Vc and C1/C2, resulting in a purinergic-dependent central sensitization of Vc and C1/C2 nociceptive neurons [16,38-40]. Recently it has been found that Vc central sensitization can be produced by specifically stimulating these P2X receptors in the pulp [22].

In the present study, we observed that GG muscle activity could be evoked by pulpal application of the P2X_3,2/3 _receptor agonist α,β-meATP 100 mM and was significantly larger in the rats when compared with PBS-administration to the pulp. The GG activity was significantly reduced in the rats receiving co-application of 100 mM α,β-meATP with the P2X_3,2/3 _receptor inhibitor TNP-ATP (1 mM). A large number of pERK-LI cells were also expressed in the Vc, Vi/Vc, C1/C2 and Pa5 5 min after pulpal application of 100 mM α,β-meATP. The pERK-LI cells and GG activity were significantly reduced after intrathecal injection of the MEK inhibitor PD98059. In addition, the number of pERK-LI cells was significantly reduced in rats receiving pulpal co-application of 100 mM α,β-meATP with 1 mM TNP-ATP compared with rats receiving 100 mM α,β-meATP alone.

### Technical considerations

In the present study, dental paper points soaked with PBS, α,β-meATP or TNP-ATP were inserted into the tooth pulp. The insertion of a paper point into the tooth pulp may itself cause strong mechanical stimulation of pulpal nerve fibers. We observed some GG activity and many pERK-LI cells were expressed in the Vc, C1/C2 and Pa5 following pulpal insertion of a PBS-dipped paper point. Thus, the GG activity and pERK-LI cells expression were likely a reflection of both mechanical stimulation of the tooth pulp as well as activation of purinergic receptors when α,β-meATP was applied. Nonetheless, the use of PBS as a vehicle control allowed for separation of the effects of α,β-meATP per se. Indeed, the GG activity was significantly larger following pulpal application of 100 mM (but not 10 mM) α,β-meATP when compared with PBS application. Likewise, pERK expression was significantly larger with 100 mM α,β-meATP than PBS. The dose of α,β-meATP required was higher than that reported in other parts of the body (e.g., reduction of hind paw withdrawal threshold, hind paw lifting and licking) [41-43]. This may be due to damage of ATP receptors in tooth pulp nerve fibers during the preparation of the pulp for subsequent drug administration since it has been reported that pulpal P2X_3 _receptors are highly concentrated in the odontoblastic layer [9,12] and thus susceptible to damage by cavity preparation. This may explain why a high concentration of ATP was necessary to produce a reflex effect and ERK phosphorylation. Thus, the high concentration of ATP is needed for reflex and other effects relative to other body regions.

We could not observe significant activation of MA or DA muscle following α,β-meATP administration. Primary afferent activity is not sufficiently strong to activate MA motor neurons relative to GG muscle, following noxious mechanical stimulation of facial skin, based on our EMG recordings as described in the Results section. Furthermore, P2X receptor density is lower in the tooth pulp relative to other receptors [13]. Therefore, the tooth pulp-MA and tooth pulp-DA pathways have a higher threshold for activation by tooth pulpal administration of ATP agonists compared with the tooth pulp-GG pathway.

### Involvement of P2X receptor activation of tooth pulpal afferents

It is well known that peripheral inflammation or tissue injury causes peripheral ATP release from non-neuronal cells in the injured region [9,10,17]. The ATP released from the non-neuronal cells binds to purinergic receptors such as P2X_3 _and/or P2X_2/3 _receptors in C-fiber terminals, resulting in peripheral sensitization of the primary afferent neurons [1,10,11,17,44]. We observed strong activation of GG activity following pulpal application of α,β-meATP, indicating that activation of pulpal P2X_3 _and/or P2X_2/3 _receptors in the tooth pulp is sufficient for activation of the GG reflex. The increase in GG muscle activity with α,βme-ATP and its 1-2 sec latency are consistent with other studies of muscle (MA or DA muscle) responses evoked by tooth pulp stimulation [24] or other orofacial stimuli, eg. TMJ [36,37]. Shigenaga et al. [45,46] have reported that tooth pulp afferents project to the Vc and trigeminal spinal subnucleus interpolaris (Vi), and many Vi neurons send projection axons to GG muscle motor neurons [47]. Furthermore, previous electrophysiological studies have also reported that GG or hypoglossal motor neuron activity is modulated by trigeminal nerve stimulation, suggesting that the trigeminal afferent is involved in modulation of GG muscle activity [48,49]. Previous studies have also reported that the pulpal application of mustard oil or capsaicin induces EMG activity in the MA and DA muscles simultaneously, and these excitations could last more than 1 min [23,24]. On the other hand, pulpal application of α,β-meATP in the present study caused a significant increase in EMG activity only in the GG muscle, and the duration of GG activity was less than 1 min. These differences between the studies might be explained by different experimental conditions in the studies or that the tooth pulp afferent-GG reflex pathway especially involves P2X_3,2/3 _receptors.

Together with previous results our findings suggested that tooth pulp nerve fibers were sensitized by ATP released from the non-neuronal pulpal cells following tooth pulp inflammation or injury, resulting in the barrage of action potentials in the tooth pulp nerve fibers which were conveyed to Vc and C1/C2 neurons.

### Sensitization of Vc, C1/C2 and Pa5 neurons

It has been documented that noxious inputs from the orofacial region are somatotopically organized in the Vc complex and C1/C2 regions, and nociceptive neurons in these areas are involved in the localization of orofacial pain [50]. Anterograde tracing studies have revealed that the rat's tooth pulp afferents are distributed in the ipsilateral Vc, C1/C2 and Pa5 [45,46,51-55]. In particular, the maxillary first molar pulp afferent projects to the ipsilateral Vc, Vi/Vc, Pa5 and C1/C2 regions [54]. Shimizu et al. have also reported that pulpal application of capsaicin produces pERK-LI cell expression in these regions suggesting that neurons in Pa5, Vc, Vi/Vc and Vc/C2 regions are involved in tooth pulp nociceptive processing [23]. Consistent with these previous studies, the present study documented expression of pERK-LI cells following α,β-meATP pulpal application in the dorsal portion of the ipsilateral Vc, Vi/Vc, C1/C2 and bilaterally in the Pa5 region. It is well established that Vc, Vi/Vc and C1/C2 nociceptive neurons manifest marked central sensitization following orofacial inflammation or trigeminal nerve injury [40,56,57]. It is also known that a barrage of action potentials is elicited in primary afferent fibers following tooth pulp inflammation [58]. Peripheral inflammation is thought to be involved in enhancement of a variety of receptor activities, including purinergic receptor in peripheral nerve terminals [42], which results in the peripheral and central sensitization of the trigeminal nociceptive system [16,22]. Together, these data and the present results suggest that purinergic receptors in pulpal nerve terminals are involved in peripheral sensitization of the trigeminal nociceptive system. It has also been reported that Pa5 neurons are involved in autonomic regulation as well as in nociceptive processes [59].

ERK is one of the MAPK families, and has been documented in the spinal DH as well as dorsal root ganglion neurons that are phosphorylated by noxious peripheral stimulation in an intensity-related manner [60-64]. ERK has also be shown to be phosphorylated in Vc and C1/C2 neurons within 5 min after noxious stimulation of the orofacial region [28], strongly suggesting that ERK phosphorylation is involved in the activation of nociceptive neurons in the Vc and C1/C2 soon after orofacial noxious stimulation. Consistent with these findings, we observed that many pERK-LI cells were expressed in Vc, Vi/Vc, C1/C2 and Pa5 regions 5 min after pulpal application of α,β-meATP, and showed that the pERK-LI cells in Vc showed NeuN immunoreactivity, indicating that the phosphorylation of ERK occurred in neurons. Furthermore, following intrathecal administration of the MEK inhibitor PD 98059 [27,30,31,65], the pERK-LI cells were significantly reduced in Vc, Vi/Vc, C1/C2 and Pa5 in rats receiving pulpal administration of α,β-meATP, suggesting that the intracellular ERK cascade is involved in the activation of Vc, Vi/Vc, C1/C2 and Pa5 nociceptive neurons. We also observed that the GG activity evoked by pulpal application α,β-meATP was significantly suppressed by intrathecal administration of PD 98059. Since it has been reported that Vc neurons are involved in masticatory muscle activity evoked by noxious stimulation of orofacial tissues [66,67], the ERK phosphorylation in Vc neurons was likely involved in our documented enhancement of GG activity following pulpal administration of α,β-meATP.

## Conclusions

The present findings suggest that P2X_3 _and P2X_2/3 _receptors may be involved in the activation of tooth pulpal nerve fibers following tooth pulp injury, resulting in central sensitization of Vc, Vi/Vc, C1/C2 and Pa5 neurons through the intracellular MAP kinase cascade. The findings underscore the importance of purinergic receptor mechanisms in tooth pulp nociceptive processes.

## Competing interests

The authors declare that they have no competing interests.

## Authors' contributions

All authors read and approved the final manuscript. KA carried out the experiments and data analysis. KS, IS and MM helped the experiments, data analysis and paper writing. BJS, MS and JWH provided data interpretation and helped to finalize the manuscript. HS, KH and NK provided data interpretation. KI conceptualized the hypothesis, designed and supervised the experiments, directed the data analysis, and finalized the manuscript.
